# Actionable wastewater surveillance: application to a university residence hall during the transition between Delta and Omicron resurgences of COVID-19

**DOI:** 10.3389/fpubh.2023.1139423

**Published:** 2023-05-17

**Authors:** Ryland Corchis-Scott, Qiudi Geng, Abdul Monem Al Riahi, Amr Labak, Ana Podadera, Kenneth K. S. Ng, Lisa A. Porter, Yufeng Tong, Jess C. Dixon, Sherri Lynne Menard, Rajesh Seth, R. Michael McKay

**Affiliations:** ^1^Great Lakes Institute for Environmental Research, University of Windsor, Windsor, ON, Canada; ^2^Department of Chemistry and Biochemistry, University of Windsor, Windsor, ON, Canada; ^3^Department of Biomedical Sciences, University of Windsor, Windsor, ON, Canada; ^4^Department of Kinesiology, University of Windsor, Windsor, ON, Canada; ^5^Environmental Health and Safety, University of Windsor, Windsor, ON, Canada; ^6^Civil and Environmental Engineering, University of Windsor, Windsor, ON, Canada

**Keywords:** COVID-19, RT-qPCR, SARS-CoV-2, wastewater, public health

## Abstract

Wastewater surveillance has gained traction during the COVID-19 pandemic as an effective and non-biased means to track community infection. While most surveillance relies on samples collected at municipal wastewater treatment plants, surveillance is more actionable when samples are collected “upstream” where mitigation of transmission is tractable. This report describes the results of wastewater surveillance for SARS-CoV-2 at residence halls on a university campus aimed at preventing outbreak escalation by mitigating community spread. Another goal was to estimate fecal shedding rates of SARS-CoV-2 in a non-clinical setting. Passive sampling devices were deployed in sewer laterals originating from residence halls at a frequency of twice weekly during fall 2021 as the Delta variant of concern continued to circulate across North America. A positive detection as part of routine sampling in late November 2021 triggered daily monitoring and further isolated the signal to a single wing of one residence hall. Detection of SARS-CoV-2 within the wastewater over a period of 3 consecutive days led to a coordinated rapid antigen testing campaign targeting the residence hall occupants and the identification and isolation of infected individuals. With knowledge of the number of individuals testing positive for COVID-19, fecal shedding rates were estimated to range from 3.70 log10 gc ‧ g feces^−1^ to 5.94 log10 gc ‧ g feces^−1^. These results reinforce the efficacy of wastewater surveillance as an early indicator of infection in congregate living settings. Detections can trigger public health measures ranging from enhanced communications to targeted coordinated testing and quarantine.

## 1. Introduction

SARS-CoV-2 is the virus responsible for COVID-19 (Coronavirus Disease 2019). SARS-CoV-2 infection produces less severe illness than SARS-CoV and MERS-CoV with lower mortality (1, 2). However, the basic reproduction number (R_0_) of SARS-CoV-2 is substantially higher than previous coronavirus epidemics (1). Strong evidence of airborne transmission largely explains higher transmissibility of SARS-CoV-2 (3, 4). Additionally, asymptomatic cases of COVID-19 likely promote transmission as individuals can pass the infection without knowing they are contagious (5, 6). Testing populations widely is complicated by the fact that clinical testing is expensive and can overwhelm healthcare resources. Alternate means of ascertaining disease prevalence has emerged as an important public health goal for pandemic management.

SARS-CoV-2 can be shed in the digestive tract of infected individuals and excreted in feces (7, 8). Consequently, SARS-CoV-2 RNA can be detected in untreated wastewater (9) following collection at wastewater treatment facilities (10, 11). Correlations have been found between the amount of SARS-CoV-2 viral material in wastewater and the prevalence of disease within the community served, (12) with many instances demonstrating that wastewater surveillance may provide early warning of increases in clinical cases (12–17).

Testing the footprint of disease within an entire community can help to inform public health decision making (18). However, testing wastewater “upstream” of treatment facilities arguably produces more immediately actionable data that may be used to mitigate disease transmission (19–21). During the COVID-19 pandemic, wastewater surveillance of congregate living settings has been adopted by many universities to assess disease prevalence on campus (22–27). In this setting, it has been shown to be a cost-effective means of detecting cases among individuals in high density housing, especially in comparison with clinical testing protocols (28). Wastewater surveillance can also warn of outbreaks in other congregate living settings. This type of “upstream” monitoring has been implemented in homeless shelters (29) and in long-term care facilities (30) where early detection and mitigation of transmission is especially important as the monitored populations are more susceptible to mortality associated with COVID-19 infection (31).

Upstream sampling modalities can rely on the same methodologies employed to monitor wastewater at centralized wastewater treatment facilities where composite samples are collected by autosampler. This type of sampling does not always lend itself to upstream locations where practical considerations such as autosampler deployment and variable flows can preclude sampling. Passive samplers offer an alternative, especially in logistically challenging settings where they can detect a single case per 10,000 individuals (32). Moore Swabs are a class of passive sampling device composed of absorptive material placed in a flowing medium to continuously filter particulate material for analysis (33). Moore Swabs have been used in wastewater surveillance at broad and fine spatial resolutions (i.e., monitoring upstream and at the community level) and have been shown to be equivalent to or outperform grab and composite sampling (34–36).

In February 2021, the University of Windsor implemented a program to monitor wastewater in a single residence hall on campus. During spring 2021, wastewater surveillance likely averted a COVID-19 outbreak by detecting an infection using passive samplers and analysis by RT-qPCR. The detection led to a public health response which included a testing campaign at the residence and the eventual quarantine of an infected individual and their close contacts (20). The campus monitoring program was expanded at the beginning of the 2021 fall semester to include three residence halls. This report focuses on a second occurrence in which wastewater surveillance may have prevented an outbreak. In addition to resulting in an actionable public health response, data generated provided taxonomic resolution of the variant of SARS-CoV-2 responsible and estimation of fecal shedding rates for the variant.

## 2. Methods

### 2.1. Sample collection

Passive samplers were deployed once weekly at three campus residence halls beginning in summer 2021 to establish a baseline prior to students moving to campus. Beginning in fall 2021, sampling frequency was increased to twice weekly. Swabs passively interacted with wastewater for approximately 24 h before they were collected. Once collected, swabs were placed in sealable plastic bags and transported to the laboratory on ice for immediate processing. Samplers consisted of a feminine hygiene product (Tampax Cardboard Tampons, Regular Absorbency, Procter & Gamble, Cincinnati, OH, United States) clipped to a carabiner which was attached to the interior of the rim of a sewer cover via fishing line and a magnet. Duplicate tampons were placed within each monitored sewer lateral to increase the volume of liquid absorbed.

### 2.2. Sample processing

At the laboratory, liquids and solids were expelled manually by massaging the tampons while still in the sealed plastic bag. A mean volume of 35 mL (SD ± 10) was eluted from each swab. The liquid and suspended solids were decanted into a sterile 50 mL conical polypropylene tube and centrifuged at 4820 × *g* for 40 min at 4°C. The supernatant was collected and passed through a 0.22 μm Sterivex cartridge filter (MilliporeSigma, Burlington, MA, United States). Filters were flash frozen in liquid nitrogen and were stored in liquid nitrogen at −196°C until extraction. RNA was extracted from the filters using the AllPrep PowerViral DNA/RNA kit (Qiagen, Germantown, MD, United States) modified by addition of 5% (v/v) 2-mercaptoethanol to the lysis buffer. RNA was eluted in 50 μL of RNAse-free water.

### 2.3. RT-qPCR

Template was analyzed undiluted and diluted 1:5 with RNAse-free water to relieve PCR inhibition. RT-qPCR targeted the conserved N1 and N2 regions of the nucleocapsid (N) gene of SARS-CoV-2 (37). RT-qPCR was also performed to evaluate the levels of Pepper Mild Mottled Virus (PMMoV) within the wastewater as an indicator of human fecal matter (38–40) using primers and probes described previously (41). RT-qPCR reactions for SARS-CoV-2 contained 10 μL of 2× RT-qPCR master mix (Takyon Dry One-Step RT Probe MasterMix No Rox; Eurogentec, Liege, Belgium), 5 μL of template and the remaining 5 μL consisted of forward primer (final concentration of 300 nM), reverse primer (final concentration of 300 nM) and probe (final concentration of 150 nM). All samples were run in technical triplicates for each target assayed. The thermocycling protocol for each of the gene targets was consistent. Reverse transcription was performed for 10 min at 48°C, followed by an enzyme activation step at 95°C for 3 min and 50 cycles of denaturation and annealing/extension at 95°C for 10 s and 60°C for 45 s, respectively. This protocol was carried out using a MA6000 thermocycler (Sansure Biotech, Changsha, China). No template controls were included with each RT-qPCR run and a 7-point standard curve for SARS-CoV-2 derived from serial dilution of a synthetic RNA standard (Exact Diagnostics, Fort Worth, TX, USA) was run with each set of samples. A standard curve for the quantification of PMMoV was generated through serial dilution of a custom Gblock. All standard curves were made in RNAse- free water. No amplification was observed for process controls (extraction blanks) or in no template controls. The LOD of the N1 and N2 assays is 1 copy·μL^−1^ of template, corresponding to a greater than 95% probability of detection. LOD was determined through analysis of 20 replicate 7-point standard curves. Standards for each target met the minimum requirements from Protocol for Evaluations of RT-qPCR Performance Characteristics: Technical Guidance (slope from −3.1 to −3.6 and an R^2^ value of at least 0.98) (42).

Samples were also analyzed by RT-qPCR primer extension assay targeting the mutation D63G on the N gene, which is unique to sublineages of the Delta (B.1.617.2) variant of concern (43, 44). RNA extract was diluted 1:5 with RNase-free water and 5 μL of sample was mixed with 10 μL of 2 × RT-qPCR master mix (Eurogentec), 500 nM primers and 125 nM probe in a final reaction volume of 20 μL. Reverse transcription was performed for 10 min at 48°C, this was followed by an enzyme activation step at 95°C for 3 min, 45 cycles of denaturation and annealing/extension at 95°C for 10 s and 55°C for 45 s, respectively. Primer and probe sequences were previously described (45). To quantify the SARS-CoV-2 viral load, a standard curve was generated using a synthesized gblock DNA fragment serially diluted in RNAse-free water (Supplementary Table S1).

### 2.4. Fecal shedding calculation

Estimation of fecal shedding rates followed an approach previously described (46) adopting modifications made describing a previous outbreak on the University of Windsor campus (20). The formula used to estimate fecal shedding rate was:


$$FS=\frac{\left(VC\times Q\times h\right)}{\left(G\times I\right)}$$

where *VC* is the estimated concentration of N1 gene found in the wastewater in gene copies·L^−1^. *Q* is the approximate flow rate of water leaving the residence hall in L·min^−1^ and *h* is a constant that allows the conversion of units. In the denominator, *G* is the median *per capita* wet weight mass of feces from high income countries (47) and *I* is the number of infected individuals contributing to shedding SARS-CoV-2 viral material into the sewer. As in previous work regarding fecal shedding, the absolute gene copies·L^−1^ of N1 was calculated using the median PMMoV (2.32 × 10^6^ gene copies·L^−1^) from 17 grab samples collected in February and March, 2021 (20). This was necessary since it is challenging to produce accurate estimates of SARS-CoV-2 gene concentration in the sampled water itself using passive samplers. However, an accurate back estimation may be made using the ratio between SARS-CoV-2 and PMMoV gene concentrations found in the material collected by the passive samplers. This assumes that the passive sampling device captures PMMoV and SARS-CoV-2 with equal efficiency. Sample calculations can be found in the Supplementary Material.

Flow rates were determined by examination of the water usage within the residence as recorded by a utilities meter within the building. This method of determining the flow was necessitated by the challenges associated with mounting a flow meter within the sewer and the inconsistent flow, which was often too low to be detected by a flow meter. Since monitoring was conducted at each of the laterals associated with the building but SARS-CoV-2 was only detected in one of the two laterals, flow per resident was calculated and adjusted to reflect the number of residents housed in the north portion of the building (corresponding to the lateral that tested positive for SARS-CoV-2).

### 2.5. Ethics review

The information on the cases described are considered exempt from ethics review under the Canadian *Tri-Council Policy Statement: Ethical Conduct for Research Involving Humans – TCPS 2 (2018)* Articles 2.4 and 2.5.

## 3. Results and discussion

### 3.1. Campus wastewater surveillance

During summer 2021, student residence halls at the University were minimally occupied with ~30 students residing in one building. The occupied building was monitored once weekly over the summer semester with no detections of SARS-CoV-2. The University opened 3 residence halls (hereafter referred to as Residence A, Residence B and Residence C) in late August 2021. In preparation for the resumption of occupancy for these 3 buildings, frequency of wastewater monitoring was increased to twice weekly the week before students resumed occupancy. A total of 526 students inhabited the 3 residence halls at the beginning of the fall semester. As part of the University’s Return to Campus initiative, students living in residence halls were required to have at least 1 dose of a vaccine approved by Health Canada.

**Figure 1:**
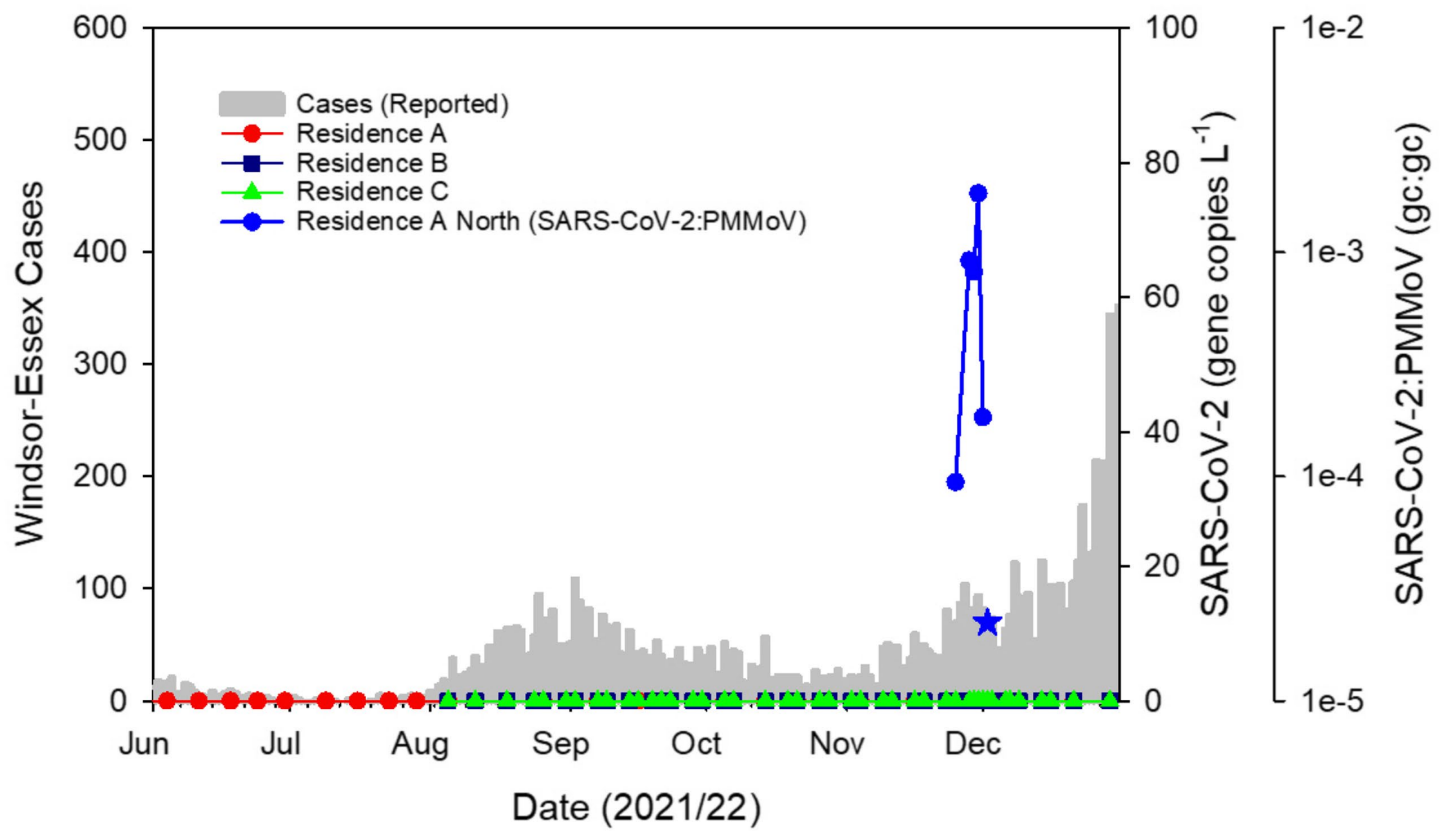
SARS-CoV-2 in campus residence hall wastewater plotted as the ratio of gene copies (gc) of SARS-CoV-2:PMMoV against COVID-19 cases in the Windsor-Essex region by reported date. Sampling the wastewater at 3 residence halls with Moore swabs twice weekly over a 12-week period showed no detectable SARS-CoV-2 following which detection related to the outbreak described here commenced with a sample collected on November 25, 2021. SARS-CoV-2 remained detectable through December 2 albeit yielding a weak signal on that date (blue star) and RT-qPCR amplification of only two technical replicates, both yielding Ct values outside the range of the standard curve. Thereafter, SARS-CoV-2 was not detected in campus residence sewer laterals through the remainder of the fall semester.

Wastewater testing yielded no detections of SARS-CoV-2 at residence halls through the beginning of the semester (Figure 1). This trajectory mirrored the low incidence of COVID-19 in the Windsor-Essex region at this time (Supplementary Figure S1). It was likewise consistent with low concentration of SARS-CoV-2 detected in municipal wastewater following an August–September 2021 resurgence due to the Delta variant of concern (VOC) (Supplementary Figure S1). Also contributing to the apparent absence of disease on campus was a relatively low student population housed in residence halls combined with suspension of most in person classes during fall semester. Additionally, the University’s vaccination policy for on-campus students likely helped to reduce the chance of an outbreak prior to the November–December infections detailed in this report. Given the regularity in which monitoring was conducted and the duration of passive sampler deployment, it is unlikely that surveillance efforts during the fall semester failed to capture cases of COVID-19 within the residence halls monitored.

Following nearly 3 months of non-detects for SARS-CoV-2, on November 26, 2021, a wastewater sample collected at Residence A tested positive with triplicate technical replicates for the N1 gene region yielding a mean pepper biomarker normalized ratio of 9.4 × 10^−5^ (±1.4 × 10^−5^SD; Figure 1). The detection triggered daily sampling of each residence hall and prompted separate sampling of sewer laterals serving distinct wings of Residence A allowing spatial isolation of SARS-CoV-2 signal. Subsequent samples collected from Residence B, Residence C and Residence A South sewer lateral showed no SARS-CoV-2 signal (Figure 1). The initial detect of SARS-CoV-2 was followed by a weak signal from a sample collected the following day where only a single technical replicate amplified for the SARS-CoV-2 N1 gene target. The reasons for this weak signal are unknown but the concentration of fecal biomarker from this sample was also low suggesting that the sample was dilute or that inhibition was present within the wastewater matrix. Alternatively, the initial infected individual(s) may not have contributed to the wastewater sampled either due to irregular defecation patterns (48) and the weak signal caused by residual SARS-CoV-2 material within the sewer lateral. Sample placement, timing and duration are important considerations for accurate monitoring (49). Whatever the reason for the weak detect, this signal invited the possibility that the initial detect was caused by a transient visitor rather than an occupant. Therefore, public health action was paused while a third sample was collected. Passive samplers placed on November 27 were collected the following day. Residence B, Residence C and Residence A South showed no signs of COVID-19 infections. However, the sample collected from Residence A North yielded a robust SARS-CoV-2 signal with a mean pepper-normalized SARS-CoV-2 ratio of 9.2 × 10^−4^ (±3.6 × 10^−5^ SD), one order of magnitude higher than the initial detect (Figure 1). The lower Ct values associated with this sample as well as the persistence of the signal over 3 days led to the conclusion that an individual within the building was likely infected with COVID-19. This information was communicated to the University leadership and daily sampling was continued to achieve high temporal resolution monitoring of the sewage leaving the residence hall. A sample collected on November 30 showed a continued upward trend in SARS-CoV-2 signal intensity at Residence A North (N1:PMMoV mean 1.8 × 10^−3^ ± 5.9 × 10^−4^) with the biomarker-normalized SARS-CoV-2 ratio having increased by two orders of magnitude over the initial detection (Figure 1). Viral signal was absent at all other monitored sites. Daily testing of the wastewater at all residence halls on campus continued for the next 3 days. The signal at Residence A North waned rapidly to levels that were undetectable by December 3. SARS-CoV-2 was not detected in campus wastewater for the remainder of the semester.

Wastewater monitoring at upstream sites may act as mirrors of trends within the community (Supplementary Figure S2) (50, 51). Additionally, variant-specific assays as well as sequencing of variants within upstream sites can provide insight about variants of SARS-CoV-2 within the larger population. It is easier to resolve variants in upstream sewage than sewage from community level wastewater treatment plants since fewer individuals contribute to the signal. During the outbreak on campus, the Delta VOC was dominant within the southwest region of Ontario as confirmed by variant-specific RT-qPCR (Supplementary Figure S1), as well as wastewater sequencing and sequencing of a subset of clinically confirmed cases (52). The Delta VOC was characterized by increased transmissibility, higher replication efficiency and viral loads, shorter incubation times and vaccine evasion (53–55). RT-qPCR analysis conducted on RNA extracted from the wastewater sample collected at Residence A North on November 30 showed evidence of the presence of the D63G mutation in the N gene which is diagnostic of the Delta VOC (43, 44). Next-Generation sequencing of this sample confirmed that the strain responsible for the outbreak was likely the Delta sublineage AY.103 (Supplementary Data). Province-wide, this sublineage represented ~20% of reported cases based on sequencing of clinical samples between epi weeks 45–48 (November 7 to December 4, 2021) only trailing sublineage AY.25 as the dominant circulating strain in the province (56). However, within the Windsor-Essex region, sublineage AY.103 was dominant, accounting for 47.9% of the 885 cases reported by the health unit over this same period (56).

Wastewater surveillance also facilitated estimates of fecal shedding rates calculated based on the ratio between gene copies of N1 and PMMoV for each day of the outbreak. Shedding rates were calculated for each sample and with different assumed numbers of infected individuals contributing to the SARS-CoV-2 signal (Table 1). Rates of shedding increased over the first 5 days of the outbreak, likely corresponding to progression in infection and/or new infections. This is consistent with literature indicating that viral shedding peaks 4–6 days following infection, coincident with symptom onset (57–60). In this study we report fecal shedding rates ranging from 3.70 log10 gc ‧ g feces^−1^ to 5.94 log10 gc ‧ g feces^−1^. This range is lower than expected given reports of higher viral titres for the Delta VOC (53–55) but it is similar to what was calculated in a previous outbreak on the University campus that was attributed to the Alpha VOC (3.93 log10 gc ‧ g feces^−1^ to 5.99 log10 gc ‧ g feces^−1^) (20). The reported maximum of 5.94 log10 gc‧g feces^−1^ likely represents a maximal or near maximal viral load as it was estimated approximately 5 days after the initial SARS-CoV-2 detection (54, 55) and was the peak level measured in the wastewater stream. Estimates of fecal shedding rates were indirectly ascertained based on the ratio of SARS-CoV-2:PMMoV (Supplementary Table S3) in wastewater concentrated from a passive sampling device and must be cautiously interpreted. Further uncertainty in the estimate may be attributed to the flow rates used in the calculation being estimated based on facility water usage. However, estimated fecal shedding rates largely fall within the range produced by direct measurement of stool samples of COVID-19 patients reported in select recent studies, supporting the validity of approximation methods (Supplementary Table S4). Finally, multiple studies have shown that not all infected individuals shed SARS-CoV-2 RNA in stool (58, 61).

**Table 1:** Calculation of fecal shedding rates.

	Fecal shedding rate (log10 gc ‧ g feces^−1^)									
	Persons infected									
Date (2021)	1	2	3	4	5	6	7	8	9	10
11–28	5.62	5.32	5.14	5.02	4.92	4.84	4.78	4.72	4.67	4.62
11–29	5.55	5.25	5.07	4.95	4.85	4.77	4.70	4.64	4.59	4.55
11–30	5.94	5.64	5.46	5.34	5.24	5.16	5.09	5.03	4.98	4.94
12–01	4.93	4.63	4.45	4.33	4.23	4.15	4.09	4.03	3.98	3.93
12–02	4.30	4.00	3.83	3.70	3.60	3.52	3.46	3.40	3.35	3.30

A range of rates were calculated taking into consideration the possible number of infected individuals who contributed to the wastewater stream during this outbreak. Given the immediate rapid decline of wastewater signal following the removal of the 4 infected individuals identified with rapid tests, it is likely that the number of infected individuals contributing to the signal was no larger than 4. In addition, rates were calculated for each day of the outbreak based on the change in SARS-CoV-2 signal intensity.

The vaccination status of the cases in the present outbreak is unknown but should not influence the viral concentration as the viral loads for vaccinated and unvaccinated individuals infected with the Delta VOC are similar (60, 62, 63). Understanding fecal shedding rates in a more controlled congregate living setting can allow for better interpretation of community level wastewater surveillance data especially in estimating the number of cases associated with the catchment of a particular wastewater treatment plant. This application is especially important as wide-scale clinical testing waned during the emergence of the Omicron VOC, and public health has become more reliant on wastewater data to track the progression of the pandemic (52). Hence, using small scale studies to determine fecal shedding rates may aid in more accurate estimation of SARS-CoV-2 caseloads in the community (64, 65). Some studies have attempted to quantify SARS-CoV-2 shedding rates with a top down approach by using the number of reported cases within a population and back calculating fecal shedding rates by considering the SARS-CoV-2 gene concentrations within the wastewater collected at treatment facilities (66). However, these attempts rely on the assumption that case counts are accurate and do not properly account for loss in signal within the sewershed from adsorption to solids, oxidation and microbial activity (67). Thus, outbreaks in upstream monitoring locations offer better opportunities to calculate fecal shedding rates.

### 3.2. Public health response

All campus residence hall occupants were messaged on the evening of November 27 notifying them of the positive wastewater results at Residence A and reinforcing University COVID-19 protocols including health self-assessments, physical distancing, hand washing and mask wearing. They were also asked to refrain from receiving visitors from other residence halls. With continued positive wastewater results from Residence A North, residents of this hall were again messaged the morning of November 29 encouraging residents to avail themselves of on-campus COVID-19 rapid testing and informing them of the temporary closure of common areas in the building. The campus testing center received 35 students on November 30 of which one occupant of Residence A North tested positive and was moved, along with a close contact, to a quarantine floor in a separate building by late afternoon. Two additional students reported positive tests on December 1 and were relocated to quarantine by noon that day. Testing was moved on-site at Residence A on December 1 to attract more students for testing but resulted in only 2 additional students submitting to rapid testing. Also on December 1, the Office of Health and Safety issued an update to the University community alerting all students, faculty and staff to the evolving situation. On December 2, an additional positive case was reported who along with 3 close contacts was relocated to quarantine. On December 4, the University released a press statement indicating that a total of 4 cases had been detected and identified wastewater surveillance as the main indicator that triggered the identification of the cases (68). Several close contacts voluntarily isolated off campus and their infection status is unknown. No additional cases of COVID-19 were reported among student residents through to the end of fall semester in contrast to the winter 2022 semester (Supplementary Figure S2).

Rapid communication of monitoring data is critical in using wastewater-based surveillance as a tool to mitigate spread of COVID-19. A wastewater monitoring program implemented by the University of California San Diego focused on high frequency testing and rapid information dissemination to diagnose an estimated 85% of COVID-19 cases on campus early in the course of the disease (69). The authors stressed the importance of timely reporting and coordination between wastewater surveillance campaigns and clinical testing efforts, an opinion echoed across upstream monitoring programs (20, 70). In the present case, once it was confirmed that the SARS-CoV-2 detected within the sewer lateral for Residence A was not an anomaly, action was taken by the University in consultation with the local public health unit. Messaging targeting building occupants encouraged voluntary testing and reinforced COVID-19 protections and protocols in effect at the University. Only ~10% of the Residence A occupants submitted to rapid antigen tests administered on-site. In contrast, a similar incident on the University campus the previous spring resulted in a much higher uptake of testing (27). The lower uptake reported here may be related to pandemic fatigue as adherence to transmission mitigation policies is prone to decline over time (71, 72). Despite the lower test uptake, this study represents the successful implementation of wastewater-based surveillance in coordination with clinical testing to reduce the impact of an outbreak. Without the application of wastewater surveillance, these cases may have infected others on campus and within the larger community (73). Here we demonstrate that wastewater-based surveillance at fine spatial resolution can produce actionable data.

If the population under surveillance is informed about wastewater monitoring and trusts the results, clinical testing may not always be necessary to prevent spread. Instead, promoting awareness of the likely presence of COVID-19 infections and advising the adoption of transmission mitigating practices may be enough to curtail outbreaks. In fact, because of monitoring efforts on campus, signs are now posted within each of the monitored residence halls to inform students of wastewater results. Signs are updated on a weekly basis, are color-coded for easy interpretation, and are designed to encourage behaviors that reduce the likelihood of the transmission of respiratory infections. Continued challenges in the use of wastewater surveillance include variability in the wastewater matrix leading to quantification issues, ensuring continued buy-in from administrators, residents, and public health agencies as well as convalescent shedding that can obscure the relevancy of signals (especially in larger congregate living settings where recovering cases and new infections cohabitate) (74). Despite these challenges, wastewater-based surveillance for monitoring respiratory and other transmissible infections in congregate living settings is a promising direction that can produce highly actionable data for public health agencies and other administrations responsible for the health of residents. Possible extensions of SARS-CoV-2 surveillance include use of these methodologies to monitor other respiratory pathogens such as Respiratory Syncytial Virus (RSV) and Influenza (75, 76).

## Data availability statement

The datasets presented in this study can be found in online repositories. The names of the repository/repositories and accession number(s) can be found at: https://www.ncbi.nlm.nih.gov/genbank/, OQ180905.

## Author contributions

RC-S: conceptualization, methodology, validation, formal analysis, investigation, and writing- original draft preparation. QG: methodology, validation, formal analysis, investigation, data curation, and writing- reviewing and editing. AA and AL: investigation. AP: formal analysis, investigation, data curation, and writing- reviewing and editing. KN: formal analysis, investigation, resources, writing- reviewing and editing, supervision, and project administration. LP: writing- reviewing and editing, supervision, and project administration. YT, SM, and JD: resources, writing- reviewing and editing, supervision, and project administration. RS: conceptualization, methodology, writing- reviewing and editing, and supervision. RM: conceptualization, resources, writing- reviewing and editing, visualization, supervision, project administration, and funding acquisition. All authors contributed to the article and approved the submitted version.

## Funding

Funding in support of the Ontario Wastewater Surveillance Initiative was provided by the Ontario Ministry of Environment, Conservation and Parks. AA and AL were supported by the University of Windsor Outstanding Scholars Program.

## Conflict of interest

The authors declare that the research was conducted in the absence of any commercial or financial relationships that could be construed as a potential conflict of interest.

## Publisher’s note

All claims expressed in this article are solely those of the authors and do not necessarily represent those of their affiliated organizations, or those of the publisher, the editors and the reviewers. Any product that may be evaluated in this article, or claim that may be made by its manufacturer, is not guaranteed or endorsed by the publisher.
