# Supplementary material for: Actionable wastewater surveillance: application to a university residence hall during the transition between Delta and Omicron resurgences of COVID-19
Source: Front Public Health. 2023 May 17;11:1139423. doi: 10.3389/fpubh.2023.1139423 (PMC10230041; doi:10.3389/fpubh.2023.1139423)
Supplement: Supplementary file 1 [file Data_Sheet_1.pdf]

Supplementary Material

Actionable wastewater surveillance: application to a university residence hall during the transition between Delta and Omicron resurgences of COVID-19

Ryland Corchis-Scott, Qiudi Geng, Abdul Monem Al Riahi, Amr Labak, Ana Maria Podadera Gonzalez, Kenneth K.S. Ng, Lisa A. Porter, Yufeng Tong, Jess C. Dixon, Sherri Lynne Menard, Rajesh Seth and R. Michael McKay*

*** Correspondence: R. Michael McKay: Robert.McKay@uwindsor.ca**

1. Supplementary Methods

1.1 Sequencing and Bioinformatics

Library preparation was performed employing the NEBNext ARTIC SARS-CoV-2 Companion Kit (Oxford Nanopore Technologies, Oxford, UK) following the nCoV-2019 sequencing protocol v3 (1) with modifications. For reverse-transcription, 500 ng of RNA in 16 µL were mixed with 4 µL of LunaScript RT SuperMix (New England Biolabs, Ipswich, MA, USA) and incubated at 25 °C for 5 min followed by an incubation at 55 °C for 20 min. For cDNA amplification, the SARS-CoV-2 specific version 3 primer set designed by ARTIC Network was used. Multiplex-polymerase chain reaction was performed for each of both SARS-CoV-2 primer pools mixing 7.5 µL of Q5® Hot Start High-Fidelity 2× Master Mix and 5.5 µL of the cDNA template with either 2 µL of primer pool #1 or primer pool #2. PCR reactions were carried out in duplicate. The mix was incubated at 98 °C for 30 sec followed by 35 cycles at 95 °C for 15 sec and 63 °C for 5 min before holding at 4 °C. PCR products were pooled and cleaned by adding 1 volume of NEBNext Sample Purification Beads (New England Biolabs). Sample was eluted in 15 µL nuclease-free water and quantified using a spectrophotometer (DS-11; DeNovix Inc., Wilmington, DE, USA). The end-prep reaction was performed employing 200 ng of PCR product mixed with 2.3 µL Ultra II End Prep Buffer (New England Biolabs), 1 µL Ultra II End Prep Enzyme (New England Biolabs) and water to a final volume of 20 µL. The reaction was incubated at 20 °C for 10 min followed by 65 °C for 10 min and holding at 4 °C. Barcoding of samples was carried out combining 3 µL of end-prepped sample, 2.5 µL Native Barcode (native barcoding EXP-NBD104, Oxford Nanopore Technologies), 10 µL Blunt/TA Ligase Master Mix (New England Biolabs) and 4.5 µL nuclease-free water. The ligation reaction was incubated at 22 °C for 20 min followed by 65 °C for 10 min and a hold on ice for at least 1 min. Cleaning of the ligated sample was performed by adding 0.4 volume NEBNext® Sample Purification Beads (New England Biolabs) and eluting with 12 µL of nuclease-free water. Oxford Nanopore sequencing adaptor ligation was performed employing 200 ng of barcoded DNA in 30 µL, mixed with 5 µL Adapter Mix II, 10 µL 5× NEBNext Quick Ligation Reaction Buffer (New England Biolabs) and 5 µL Quick T4 DNA Ligase (New England Biolabs). The incubation was carried out at 25 °C for 20 min. Sample was cleaned by adding 1 volume of NEBNext Sample Purification Beads

(New England Biolabs), eluted in 12 µL of elution buffer and quantified. Twenty ng of the library was loaded onto a minION Spot-ON Flow Cell (Oxford Nanopore Technologies).

A modified version of the bioinformatic pipeline available from Galaxy server (2) "*COVID-19: variation analysis of ARTIC ONT data"* was employed to analyze sequences. This pipeline facilitated rapid provision of pre-process sequences from FASTQ files. Mapping reads was carried out with minimap2 tool. Samtools, iVar trim and QualiMap BamQC were employed to convert between alignment formats, trim primer sequences and evaluate the quality of mapped reads, respectively. Medaka consensus and medaka variant tools were used to create a consensus sequence from Nanopore sequencing data and decode variant calls. Genetic variant annotation and effect prediction were carried out through bcftools and SnpEff toolbox. Consensus sequence generated by applying VCF variants (bcftools consensus) were further uploaded into the Pangolin web application to determine the dominant variant in the sample (3).

1. Quick J. nCoV-2019 sequencing protocol v3 (LoCost) [Internet]. protocols.io. 2020 [cited 2022 Nov 23]. Available from: https://www.protocols.io/view/ncov-2019-sequencing-protocol-v3-locost-bh42j8ye

2. Afgan E, Baker D, Batut B, van den Beek M, Bouvier D, Cech M, et al. The Galaxy platform for accessible, reproducible and collaborative biomedical analyses: 2018 update. Nucleic Acids Res. 2018 Jul 2;46(W1):W537–44.

3. O'Toole Á, Scher E, Underwood A, Jackson B, Hill V, McCrone JT, et al. Assignment of epidemiological lineages in an emerging pandemic using the pangolin tool. Virus Evolution. 2021 Dec 1;7(2):veab064.

2. Supplementary Figures and Tables

2.1 Supplementary Figures

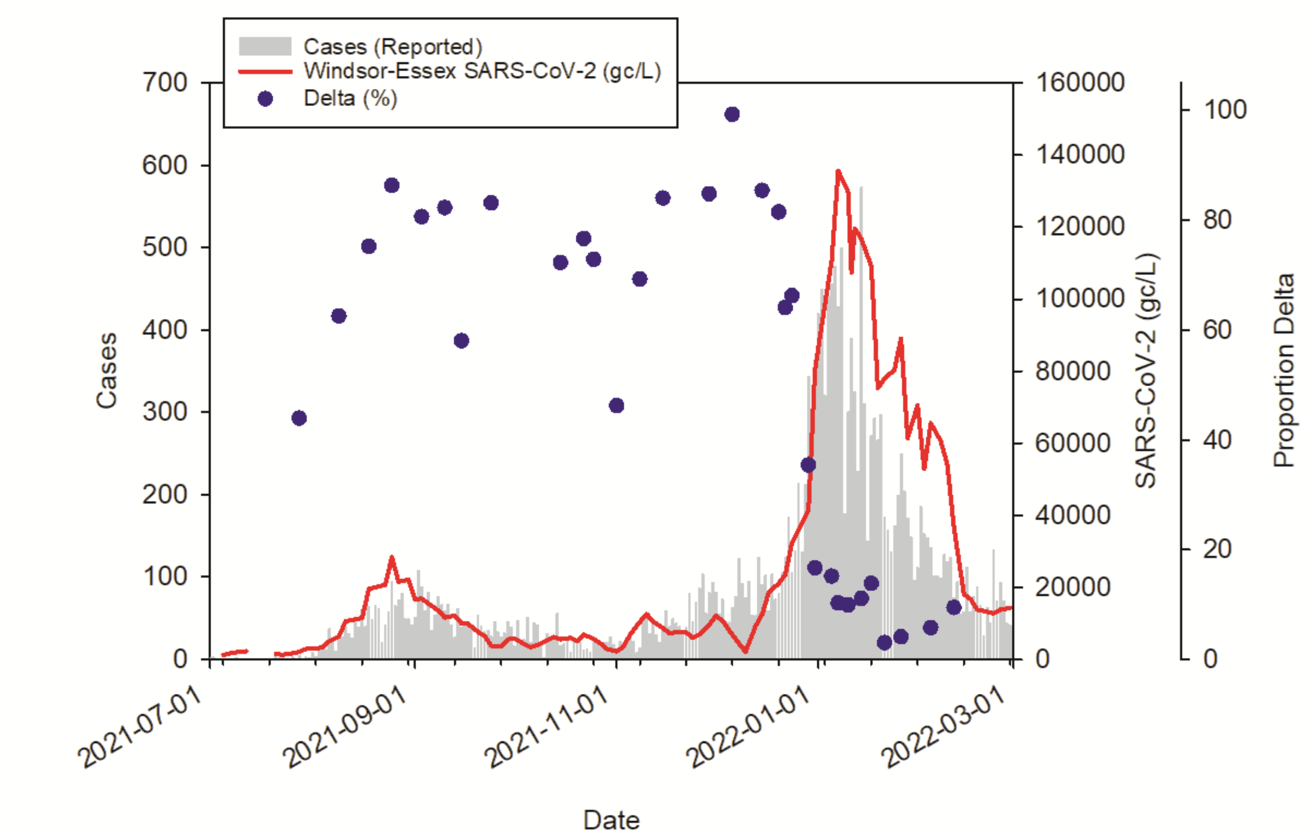

Supplementary Figure 1. Cases reported for Windsor-Essex (grey bars) spanning COVID-19 resurgences fueled by Delta (August-September 2021) and Omicron (December 2021 – February 2022) Variants of Concern (VOC) that occurred over the course of this study. Concentration of SARS-CoV-2 N1 gene target in wastewater is superimposed on the COVID-19 cases. N1 gene concentration is a 7-day running mean of aggregate data from three WWTPs in Windsor-Essex with data weighted by population served (red line). These three WWTPs treat wastewater accounting for 71% of the regional population. As part of the Ontario Wastewater Surveillance Initiative, 24-h composite samples from these plants are collected at a frequency of thrice weekly. Samples were processed the same day as collection as described previously for grab samples from residence hall wastewater (4). Occurrence of the Delta VOC was determined by N-gene primer extension assay targeting the D63G mutation as described in the Methods. RT-qPCR assays for the Delta VOC were carried out 1-2 × weekly. By late-December, 2021, the proportion of Delta VOC detected in wastewater samples plummeted as the Omicron VOC rapidly became the dominant strain circulating through the Province (5,6)

4. Corchis-Scott R, Geng Q, Seth R, Ray R, Beg M, Biswas N, et al. Averting an Outbreak of SARS-CoV-2 in a University Residence Hall through Wastewater Surveillance. Microbiology Spectrum. 2021 Oct 6;9(2):e00792-21.

5. Arts E, Brown S, Bulir D, Charles T, DeGroot C, Delatolla R, et al. Community Surveillance of Omicron in Ontario: Wastewater-based Epidemiology Comes of Age [Internet]. 2022 [cited 2022 Dec 19]. Available from: https://europepmc.org/article/PPR/PPR468647

6. Lawal OU, Zhang L, Parreira VR, Brown RS, Chettleburgh C, Dannah N, et al. Metagenomics of Wastewater Influent from Wastewater Treatment Facilities across Ontario in the Era of Emerging SARS-CoV-2 Variants of Concern. Microbiol Resour Announc. 11(7):e00362-22.

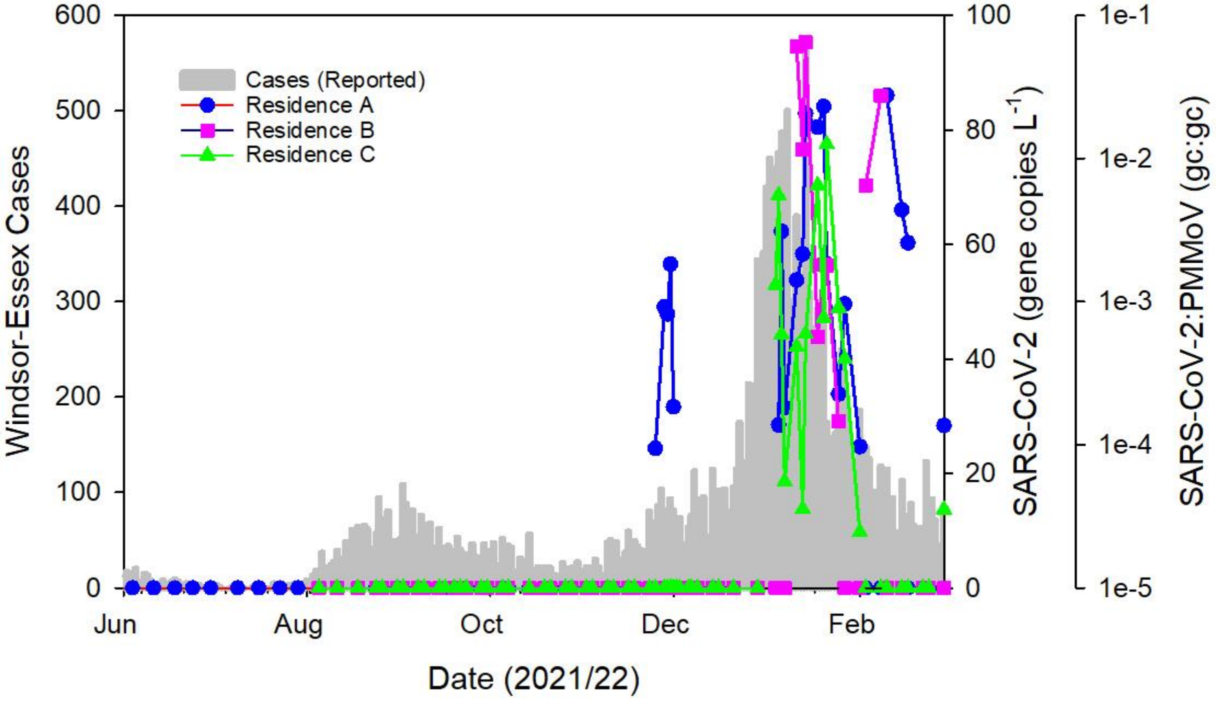

Supplementary Figure 2. SARS-CoV-2 in campus residence hall wastewater plotted as the ratio of gene copies (gc) of SARS-CoV-2:PMMoV with COVID-19 cases in the Windsor-Essex region by reported date(grey bars). Monitoring the wastewater at residence halls with passive samplers over a 3-month period yielded no detectable SARS-CoV-2 signal. The initial detection of SARS-CoV-2 related to the outbreak described here originated with a sample collected on November 25, 2021. Results from continued monitoring on campus over the emergence of Omicron highlight the challenges of data interpretation for wastewater surveillance for disease mitigation when disease prevalence is high and shows how upstream monitoring locations may act as a microcosm of the community within which they are located.

2.2 Supplementary Tables

Supplementary Table 1. Primers and probe sequences for RT-qPCR

Assay	Primers/probe	Sequence (5′-3′)	Final Conc.
N1 gene<br><br>SARS-CoV-2	N1-F	GAC CCC AAA ATC AGC GAA AT	0.3nM
	N1-R	TCT GGT TAC TGC CAG TTG AAT CTG	0.3nM
	N1-Probe	FAM-ACC CCG CAT TAC GTT TGG TGG ACC-MGBNFQ	0.15nM
N2 gene<br><br>SARS-CoV-2	N2-F	TTA CAA ACA TTG GCC GCA AA	0.3nM
	N2-R	GCG CGA CAT TCC GAA GAA	0.3nM
	N2-Probe	FAM-ACA ATT TGC CCC CAG CGC TTC AG-MGBNFQ	0.15nM
PMMoV	PMMoV-F	GAG TGG TTT GAC CTT AAC GTT TGA	0.2nM
	PMMoV-R	TTG TCG GTT GCA ATG CAA GT	0.2nM
	PMMoV-Probe	Cy5-CCT ACC GAA/TAO/G CAA ATG-3IAbRQSp	0.2nM

Supplementary Table 2. Primers, probe and gblock sequences for N-gene primer extension RT-qPCR

N:D63G Assay		N:63-Universal Assay	
D63G-F	TCACTCAACATGGCAAGAAAGG	N63 Universal-F	CTCACTCAACATGGCAAGAAAG
D63G-R	GGTAGTAGCCAATTTGGTCATCT	D63G-R	(Same as D63G Assay)
D63G-Probe	CCTTAAATTCCCTCGATGACAAGGCG	D63G-Probe	(Same as D63G Assay)

Standard gblock sequence:

D63G_N_119-313: GTC GGC CCC AAG GTT TAC CCA ATA ATA CTG CGT CTT GGT TCA CCG CTC TCA CTC AAC ATG GCA AGG AAG GCC TTA AAT TCC CTC GAG GAC AAG GCG TTC CAA TTA ACA CCA ATA GCA GTC CAG ATG ACC AAA TTG GCT ACT ACC GAA GAG CTA CCA GAC GAA TTC GTG GTG GTG ACG GTA AAA TGA AAG ATC TCA

Supplementary Table 3. Concentrations of SARS-CoV-2 and PMMoV in concentrated material expunged from passive samplers and corresponding SARS-CoV-2:PMMoV ratios used in estimation of fecal shedding rates. The highlighted row is used in the fecal shedding sample calculation

Date (2021)	N1(gc/L)	PMMoV(gc/L)	N1:PMMoV(gc/gc)
11-28	2.97×10^{4}	3.23×10^{7}	9.19×10^{-4}
11-29	3.27×10^{4}	3.99×10^{7}	8.20×10^{-4}
11-30	6.14×10^{4}	3.35×10^{7}	1.83×10^{-3}
12-01	1.95×10^{4}	1.06×10^{8}	1.84×10^{-4}
12-02	1.21×10^{3}	2.80×10^{7}	4.31×10^{-5}

Supplementary Table 4. Direct measures of SARS-CoV-2 viral load in feces found in the literature compared to estimates produced in this study. Viral RNA per microliter of eluate extracted from passive samplers is also compared to viral RNA per microliter of eluate extracted from feces (7). As might be expected, the concentration of SARS-CoV-2 RNA extracted from the passive sampler was generally lower than what was directly extracted from feces. Conversion of fecal shedding estimations from gc/g to gc/mL using a fecal density of 1.06 g/mL allows for comparison with studies that directly measured SARS-CoV-2 viral loads in feces (8). Estimated fecal shedding rates largely fall within the range produced by direct measurement of stool samples of COVID-19 patients reported in select recent studies, supporting the validity of estimation methods (9).

	Direct measure of SARS-CoV-2 viral load in feces			Estimates produced in this study			
Sample Type	Low	High	Median	Low	High	Comparison	Ref
Eluate (cp/µL)	0.32log10	3.97log10	NA	-0.68log10	1.63log10	Overlapping -Low	(7)
Stool (cp/mL)	1.63×10^{0}	4.45×10^{6}	1.01×10^{3}	5.32×10^{3}	9.18×10^{5}	Within range	(9)
Stool (cp/mL)	106.3	5.6×10^{3}	NA	5.32×10^{3}	9.18×10^{5}	Within range	(10)
Stool (cp/mL)	NA	NA	5.1 log10	3.72log10	5.96log10	Within range	(11)
Stool-Diarrhea (cp/mL)	NA	NA	3.9 log10	3.72log10	5.96log10	Within range	(11)
Stool (cp/mL)	5.50×10^{2}	1.21×10^{5}	NA	5.32×10^{3}	9.18×10^{5}	Overlapping	(12)

7. Natarajan A, Zlitni S, Brooks EF, Vance SE, Dahlen A, Hedlin H, et al. Gastrointestinal symptoms and fecal shedding of SARS-CoV-2 RNA suggest prolonged gastrointestinal infection. Med. 2022;3(6):371-387.e9.

8. Penn R, Ward BJ, Strande L, Maurer M. Review of synthetic human faeces and faecal sludge for sanitation and wastewater research. Water Research. 2018 Apr;132:222–40.

9. Daou M, Kannout H, Khalili M, Almarei M, Alhashami M, Alhalwachi Z, et al. Analysis of SARS-CoV-2 viral loads in stool samples and nasopharyngeal swabs from COVID-19 patients in the United Arab Emirates. PLoS One. 2022 Sep 22;17(9):e0274961.

10. Zhang N, Gong Y, Meng F, Shi Y, Wang J, Mao P, et al. Comparative study on virus shedding patterns in nasopharyngeal and fecal specimens of COVID-19 patients. Sci China Life Sci. 2021 Mar 1;64(3):486–8.

11. Cheung KS, Hung IFN, Chan PPY, Lung KC, Tso E, Liu R, et al. Gastrointestinal Manifestations of SARS-CoV-2 Infection and Virus Load in Fecal Samples From a Hong Kong Cohort: Systematic Review and Meta-analysis. Gastroenterology. 2020 Jul;159(1):81–95.

12. Pan Y, Zhang D, Yang P, Poon LLM, Wang Q. Viral load of SARS-CoV-2 in clinical samples. The Lancet Infectious Diseases. 2020 Apr 1;20(4):411–2.

3. Supplementary Calculations

3.1 Fecal Shedding Estimation

Sample calculation showing how fecal shedding rates were estimated. Calculations are for the passive sampler collected on November 30, 2021, and assume that 4 infected individuals were contributing to the signal. *VC* is the estimated concentration of N1 gene found in residence hall wastewater in gene copies L^{-1}. *S* is the concentration of SARS-CoV-2 N1 gene found in material expelled from the passive samples in gene copies L^{-1}. *P* is the concentration of PMMoV found in material expelled from the same passive sampler in gene copies L^{-1}. *G* is the median concentration of PMMoV from 17 grab samples collected in February and March, 2021 (2.32×10^6 gene copies L^{-1}) (4). Calculation of *VC* is as follows:

$$\boldsymbol{VC = \frac{S}{P} \times G}$$

$$\boldsymbol{VC = \frac{N1\left({}^{gc}/_{L}\right)}{PMMoV\left({}^{gc}/_{L}\right)} \times PMMoV\ Grab\ Median\left({}^{gc}/_{L}\right)}$$

$$\boldsymbol{VC = \left(\frac{6.14 \times 10^4}{3.35 \times 10^7} \times 2.32 \times 10^6\right)\left({}^{gc}/_{L}\right)}$$

$$VC = 4.24 \times 10^3 \left({}^{gc}/_{L}\right)$$

Fecal shedding calculations are as follows:

$$\boldsymbol{FS = \frac{(VC \times Q \times h)}{(G \times I)}}$$

$$\boldsymbol{FS = \frac{\left(4.24 \times 10^3\, {}^{gc}/_{L} \times 1.07\, {}^{m^3}/_{hr} \times {}^{24hr}/_{day} \times 1000\, {}^{L}/_{m^3}\right)}{(126g/person/day \times 4\ persons)}}$$

$$FS = \frac{(1.09 \times 10^8 gc)}{(504g)}$$

$$FS = 2.16 \times 10^5 \; {}^{gc}/_{g}$$

VC is the estimated concentration of N1 gene found in residence hall wastewater in gene copies L^{-1}. *Q* is the approximate flow rate of water leaving the residence hall in L min^{-1} and h is a constant that allows the conversion of units. In the denominator, *G* is the median per capita wet weight mass of feces from high income countries (13) and *I* is the number of infected individuals contributing to shedding SARS-CoV-2 viral material into the sewer.

13. Rose C, Parker A, Jefferson B, Cartmell E. The Characterization of Feces and Urine: A Review of the Literature to Inform Advanced Treatment Technology. Critical Reviews in Environmental Science and Technology. 2015 Sep 2;45(17):1827–79.
